# Thermo-Sensitive Poly (N-isopropylacrylamide-co-polyacrylamide) Hydrogel for pH-Responsive Therapeutic Delivery

**DOI:** 10.3390/polym14194128

**Published:** 2022-10-02

**Authors:** Madhappan Santhamoorthy, Thi Tuong Vy Phan, Vanaraj Ramkumar, Chaitany Jayprakash Raorane, Kokila Thirupathi, Seong-Cheol Kim

**Affiliations:** 1 School of Chemical Engineering, Yeungnam University, Gyeongsan 38541, Korea; 2 Center for Advanced Chemistry, Institute of Research and Development, Duy Tan University, 03 Quang Trung, Hai Chau, Danang 550000, Vietnam; 3 Faculty of Environmental and Chemical Engineering, Duy Tan University, 03 Quang Trung, Hai Chau, Danang 550000, Vietnam; 4 Department of Physics, Sri Moogambigai College of Arts and Science for Women, Palacode 636808, India

**Keywords:** copolymer, NIPAm, acrylamide, curcumin delivery, pH and temperature stimuli, cancer therapy

## Abstract

Stimuli-response polymeric nanoparticles have emerged as a carrier system for various types of therapeutic delivery. In this study, we prepared a dual pH- and thermo-sensitive copolymer hydrogel (HG) system (PNIPAm-co-PAAm HG), using N-isopropyl acrylamide (NIPAm) and acrylamide (AAm) as comonomers. The synthesized PNIPAm-co-PAAm HG was characterized using various instrumental characterizations. Moreover, the PNIPAm-co-PAAm HG’s thermoresponsive phase transition behavior was investigated, and the results showed that the prepared HG responds to temperature changes. In vitro drug loading and release behavior of PNIPAm-co-PAAm HG was investigated using Curcumin (Cur) as the model cargo under different pH and temperature conditions. The PNIPAm-co-PAAm HG showed pH and temperature-responsive drug release behavior and demonstrated about 65% Cur loading efficiency. A nearly complete release of the loaded Cur occurred from the PNIPAm-co-PAAm HG over 4 h at pH 5.5 and 40 °C. The cytotoxicity study was performed on a liver cancer cell line (HepG2 cells), which revealed that the prepared PNIPAm-co-PAAm HG showed good biocompatibility, suggesting that it could be applied as a drug delivery carrier. Moreover, the in vitro cytocompatibility test (MTT assay) results revealed that the PNIPAm-co-PAAm HG is biocompatible. Therefore, the PNIPAm-co-PAAm HG has the potential to be useful in the delivery of drugs in solid tumor-targeted therapy.

## 1. Introduction

Hydrogels are composed of hydrophobic and hydrophilic components that form three-dimensional polymeric network structures, which undergo sol-gel phase transition under external temperature stimuli [1]. The development of hydrogels for various biomedical applications has attracted recent research interest regarding developing novel hydrogel-based drug carriers for controlled drug delivery applications. The hydrogels are responsive to various stimuli, including temperature, light, ionic strength, etc., which are considerably significant in developing hydrogel-based nanocarrier systems for specific applications, including target delivery of various therapeutic agents [2].

The improvement of toxic therapeutics’ delivery to cancerous tissue through the use of nanocarriers as chemotherapeutic delivery systems has been noted. To increase the solubility and stability of Cur, various kinds of nanocarriers have been created [3]. Cur can be made more stable, absorbed, and delivered to target sites by being encapsulated into bio-polymeric particles or conjugated with nanoparticles, for instance [4,5,6]. Due to their outstanding benefits in terms of biocompatibility, simplicity in design and preparation, their variety of structures, and intriguing bio-mimetic properties, polymers have been extensively used as nanocarriers in recent decades [7,8,9,10].

Yocheva et al. created PDMAEMA9-PCL70-PDMAEMA9 micelles by copolymerizing poly(-caprolactone) and very short poly(2-(dimethylamino)ethyl methacrylate) segments [11]. The in vitro experimental results show an enhanced cell uptake and showed higher cytotoxicity than free Cur [12]. Tabatabaei et al. created Cur-loaded PLGA-PEG NPs and demonstrated that the anti-cancer efficacy of the prepared system was enhanced in MCF-7 cells [13]. The smart polymer hydrogels are attractive in the biological field, owing to their sharp phase transition under various stimuli, including pH, temperature, light, etc. [14,15,16,17,18,19]. Dual-stimuli responsive polymers are more frequently used in various physiological conditions than the mono-stimuli-responsive polymers [16]. Despite the fact that numerous studies have been published based on thermoresponsive PNIPAm polymers with different comonomers, including acrylic acid, N-vinylpyrrolidone, and dimethylacrylate, for a variety of applications [20], there are few reports on the copolymerization of PNIPAm with acrylamide in drug delivery applications. Therefore, in this paper, we attempted to evaluate the copolymerization of PNIPAm with acrylamide hydrogels, as well as its drug loading and release efficiency using Cur as a model drug.

Curcumin (Cur) is traditionally used in various types of therapy, owing to its beneficial ingredients with various bioactivities [21]. However, due to its low solubility and poor bioavailability, its therapeutic application is limited [22]. Cur has been discovered to have a variety of biological and anticancer activities, including antibacterial, antioxidant, and anti-inflammatory [23]. Curcumin has been studied for its ability to alleviate various cancers, including colon, and breast cancer [24]. Its anticancer and antioxidant properties have been widely used in clinical trials in recent years. Cur has high therapeutic values, but its clinical applications have been hampered by several limitations, such as low solubility, poor stability, and bioavailability. Therefore, Cur requires a specific nanocarrier system to deliver them without affecting its biological properties.

We developed a pH- and temperature-sensitive copolymer as a drug carrier in this study. The thermoresponsive polymer poly (N-isopropyl acrylamide) (PNIPAm) has caught the attention of researchers due to its sharp phase transition property. PNIPAm can change its phase transition behavior from swelling to deswelling in response to temperature changes. The drug delivery process has been optimized using PNIPAm due to its reversible phase transition property. PAAm is a stable, non-resorbable, non-toxic, and non-immunogenic copolymer based on acrylamide (AAm). To deliver Cur, PNIPAm was copolymerized with PAAm to produce the PNIPAm-co-PAAm HG. The chemical structure of PNIPAm-co-PAAm HG was investigated using ^1^H NMR, FT-IR, SEM, TEM, XPS and zeta potential analysis. The cytotoxicity test demonstrates that the PNIPAm-co-PAAm HG had biocompatibility and low cytotoxicity. In vitro drug-loading and drug-release experiments were also performed on the PNIPAm-co-PAAm HG. The zeta potential of PNIPAm-co-PAAm HG decreased as the pH increased, and the phase transition occurred at above 40 °C with concentration-dependent properties. After 4 h at pH 5.5 and 40 °C, nearly 100% of the Cur had been successfully released from the PNIPAm-co-PAAm HG matrix. The newly synthesized PNIPAm-co-PAAm HG system could release the drug in response to a physical stimulus (i.e., pH and temperature). As a result, the PNIPAm-co-PAAm HG has the potential to be useful in solid tumor-targeted therapy.

## 2. Materials

N-Isopropylacrylamide (NIPAm, 97%), acrylamide (AAm, 99%), 2,2-azobisisobutyro nitrile (AIBN, 12 wt. % in acetone), curcumin (98%), ethanol (99.5%), tetrahydrofuran (THF, 99.9%), and diethyl ether (99.7%) were purchased from Sigma Aldrich Chemical Co., Saint Louis, MO, USA, and used as received.

### 2.1. Synthesis of PNIPAm-co-PAAm Copolymer Hydrogel

**Scheme 1:**
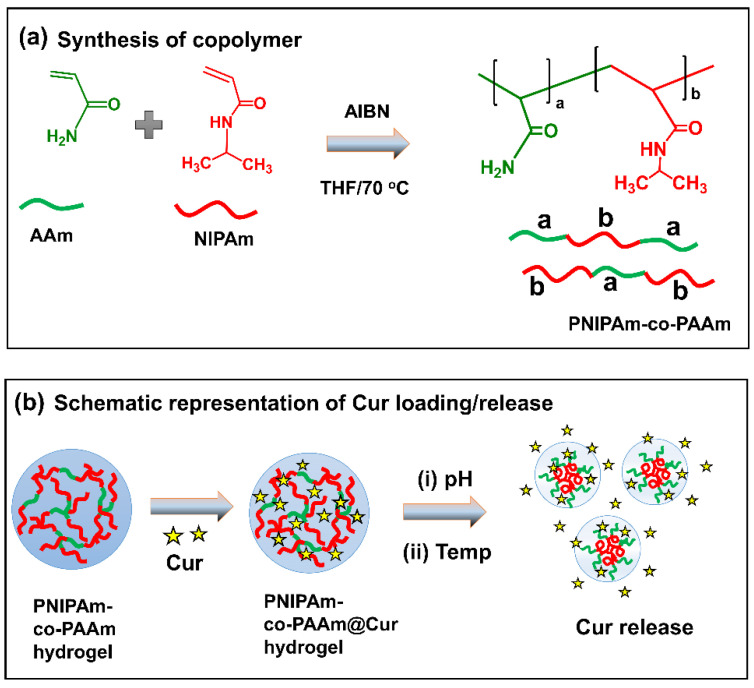
Schematic representation for the (**a**) synthesis of PNIPAm-co-PAAm HG; (**b**) curcumin loading into the PNIPAm-co-PAAm HG system, and release under the combined stimuli.

The synthesis of the PNIPAm-co-PAAm HG was performed by free radical polymerization using AIBN as an initiator [25,26]. To perform this synthesis, NIPAm (4.00 g, 35.3 mmol) and AAm (2.75 g, 35.5 mmol) were dissolved in 50 mL of two necked round bottom flasks containing 25 mL THF. Then, the reaction mixture was purged with N_2_ gas for 30 min before adding the AIBN (0.1 g in 1 mL THF) and the reaction mixture was further continuously stirred at 70 °C for 24 h. After the reaction was completed, the reaction mixture was concentrated using a rotary evaporator and the obtained viscous mass was precipitated in 100 mL of diethyl ether. The precipitation process was repeated 5 times to remove the unreacted monomers, and the obtained precipitate was dried in a vacuum oven at room temperature, overnight. The synthesized copolymer was named as PNIPAm-co-PAAm HG (Scheme 1).

### 2.2. In Vitro Cur Loading into PNIPAm-co-PAAm HG System

A hydrophobic anticancer drug Cur was chosen as a model drug for the loading and release experiments. Cur was loaded into the PNIPAm-co-PAAm HG system via swelling diffusion at a w:w ratio of 10:1 (polymer: drug) [27]. A weighed amount of dried PNIPAm-co-PAAm HG powder (0.1 g) was dissolved in 3 mL deionized water, before being mixed with 10 mg of the Cur drug. The solution was thoroughly mixed at 25 °C for 24 h. The drug encapsulated polymer sample was centrifuged at 40 °C, and the supernatant solution was collected to determine the loaded drugs into the PNIPAm-co-PAAm HG sample by using a UV–Vis spectrophotometer at 427 nm. The Cur-loaded sample was named as PNIPAm-co-PAAm@Cur (Scheme 1b). The following equations were used to calculate the percentage of Cur loaded into the PNIPAm-co-PAAm HG sample. The percentage of Cur loading was estimated to be approximately ~65%.

% Drug loading = (Wt. of the drug in sample/Wt. of sample) × 100

### 2.3. Characterization of PNIPAm-co-PAAm HG

^1^H-NMR (OXFORD, ^1^H NMR 600 MHz) was used to characterize the PNIPAm-co-PAAm PNIPAm-co-PAAm HG sample. The surface morphology and structural arrangement of the sample were observed using scanning electron microscopy (SEM, JEOL 6400 instrument, Tokyo 196-8558, JAPAN) at 10 kV accelerating voltage, and a transmission electron microscope (TEM, JEOL 2010 at 200, Tokyo 196-8558, JAPAN) at kV accelerating voltage. The elemental composition and chemical/electronic state of the copolymer were determined using X-ray photoelectron spectroscopy (XPS, Tucson, AZ 85706, USA). Fourier-transform infrared (FT-IR, JASCO FTIR 4100, Tokyo 193-0835, Japan) spectra were obtained using the KBr pelleting method. On the Malvern Zetasizer Nano-ZS, zeta potential and particle size distribution analyses of the copolymer were performed. The materials’ UV absorption spectra were recorded using an Agilent Inc. UV–VIS (Cary 60, Santa Clara, CA 95051, USA) spectrophotometer.

### 2.4. In Vitro Release of Cur from PNIPAm-co-PAAm@Cur HG

To investigate the in vitro release behavior of Cur from the PNIPAm-co-PAAm@Cur HG system, different experimental conditions were set up, including pH 7.4 and pH 5.5; 25 °C, and 40 °C, pH 7.4/40 °C and pH 5.5/40 °C, respectively. In brief, about 10 mg/mL of Cur-loaded PNIPAm-co-PAAm@Cur HG sample was placed into a dialysis bag with a molecular weight cut off of 3500 kDa. The dialysis bag was placed in a beaker containing 25 mL PBS solution, one at pH 7.4/25 °C, or pH 7.4/40 °C, respectively, and another at pH 5.5/25 °C, or pH 5.5/40 °C, respectively. To determine the amount of released Cur from the sample, about 1 mL of release medium was progressively withdrawn at prefixed time intervals and the released Cur was determined at 427 nm. The quantity of released Cur from the PNIPAm-co-PAAm@Cur HG sample was calculated using the Cur calibration curve. The following equation was used to calculate the cumulative release of Cur. Cur release (%) = (Amount of Cur release at time t / total amount of Cur in the HG taken in dialysis tube) × 100.

### 2.5. In Vitro Cytotoxicity of PNIPAm-co-PAAm HG Sample

The 3-(4,5-dimethylthiazol-2-yl)-2,5-diphenyl tetrazolium bromide (MTT) assay was performed to assess the biocompatibility of the prepared PNIPAm-co-PAAm copolymer without or with Cur loading. HepG2 cells (2 × 10^4^ cells/well) were cultured into a 96-well plate at 37 °C for 24 h. The existing medium was then replaced with a fresh medium containing various concentrations of samples such as free Cur, PNIPAm-co-PAAm HG and Cur-loaded PNIPAm-co-PAAm@Cur HG samples, respectively. After 4 h, MTT solution was added to each well and incubated for a further 4 h. Then, the DMSO (20 µL) solubilized the formazan crystals, and the absorbance was measured at 595 nm using an ELISA microplate reader.

### 2.6. Flow Cytometry (FACS) Analysis

Cell apoptosis was assayed using Muse annexin V and a dead cell assay kit (cat. no. MCH100105; BD Biosciences, Franklin Lakes, NJ, USA). The cells were harvested, washed twice with PBS, and stained with FITC annexin V and propidium iodide (PI) for 15 min at room temperature. The percentage of apoptotic cells was determined using annexin V and the Muse cell analyzer system (Merck Millipore, Burlington, MA, USA).

## 3. Results and Discussion

### 3.1. Characterization of PNIPAm-co-PAAm HG

**Figure 1:**
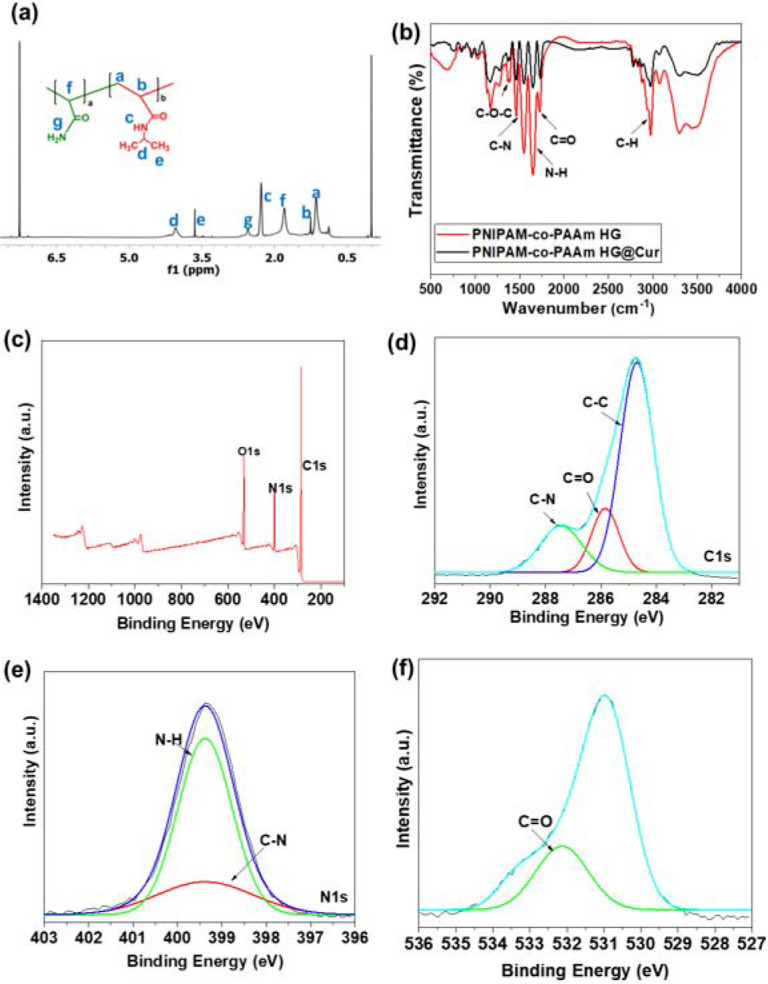
Characterization of PNIPAm-co-PAAm HG. (**a**) ^1^H NMR spectrum of PNIPAm-co-PAAm HG. (**b**) FT-IR analysis of PNIPAm-co-PAAm HG without and with Cur. XPS analysis of PNIPAm-co-PAAm HG with (**c**) wide-scan, and core level spectra of (**d**) C 1s; (**e**) N 1s; and (**f**) O 1s.

The successful synthesis of PNIPAm-co-PAAm HG was confirmed by ^1^H NMR analysis. Figure 1a depicts the ^1^H NMR spectrum of PNIPAm-co-PAAm HG, in which the appearance of the resonance peak around 1.2 ppm confirms the methyl (-CH_3_) protons. The amide peak appeared at 3.8 ppm, confirming the presence of PNIPAm segments in the copolymer chains [28]. Similarly, the primary amine –N-H signals appeared in the range between 1.9 and 2.3 ppm, depicting the incorporated PAAm segments in the PNIPAm-co-PAAm HG backbone [29]. Figure 1b depicts the copolymer’s FT-IR spectrum. The vibrational band between 2793 and 2834 cm^−1^ represented the alkyl C-H stretch of the NIPAm groups. The C=O group of two monomers was responsible for the band that appeared at 1679 cm^−1^. The stretching vibrations appeared at 1512 cm^−1^, corresponding to the N-H groups of the AAm groups. The intense band at 1376 cm^−1^ was associated with C-N stretching of the PNIPAm segments presented in the PNIPAm-co-PAAm HG system. Moreover, the Cur-loaded PNIPAm-co-PAAm@Cur HG sample showed similar stretching peaks with the PNIPAm-co-PAAm HG. However, the peak intensity slightly increased and the N-H peak slightly shifted and appeared at 1518 cm^−1^, indicating that Cur interacted with the amine groups. In addition, a new C-O-C peak appeared at 1648 cm^−1^, indicating the presence of aromatic Cur molecules [30].

The composition of PNIPAm-co-PAAm HG was studied by XPS. Figure 1c shows a wide-scan spectrum of PNIPAm-co-PAAm HG with the carbon peak (C1s) at 282–290 eV, nitrogen (N1s) at 396–403 eV, and oxygen (O1s) at 527–536 eV signals, evidencing the incorporation of the NIPAm and AAm monomers in the PNIPAm-co-PAAm HG system. High-resolution C1s spectra of PNIPAm-co-PAAm HG (Figure 1d) with a binding energy peak at 284.8 eV, attributable to the C–C of the aliphatic carbon chain, were observed. A peak at 286.5 eV for the C–N groups and a peak at 287.5 eV for the C=O groups of NIPAm and AAm monomers, respectively, were observed [31]. The deconvoluted N1s spectra (Figure 1e) clearly showed two component peaks at 398.2 eV and 399.64 eV, indicating the C-N bonds of amino groups of AAm and amide groups of NIPAm monomers [32]. Furthermore, the O1s peak position at 532.4 eV (Figure 1f) corresponds to the C=O groups of NIPAm and AAm monomers that were copolymerized in the PNIPAm-co-PAAm. The XPS spectra of the PNIPAm-co-PAAm HG confirm the successful synthesis.

**Figure 2:**
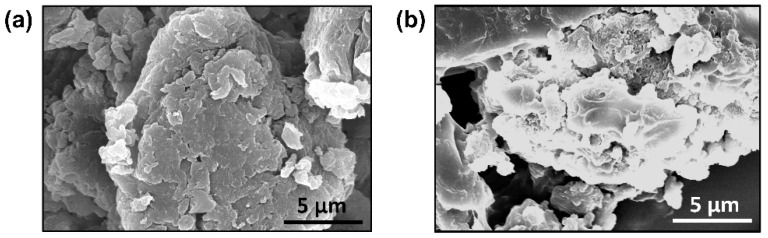
SEM images of (**a**) PNIPAm-co-PAAm HG; and (**b**) PNIPAm-co-PAAm HG@Cur samples, respectively.

The prepared PNIPAm-co-PAAm HG and PNIPAm-co-PAAm@Cur HG samples (10 mg) were dissolved in deionized water (1 mL) and the solution was heated at 40 °C to form phase separation. The obtained turbid suspension was centrifuged to separate the HG and the obtained precipitate was dried in a vacuum oven at RT to obtain the powder sample. The SEM analysis was performed with a dried PNIPAm-co-PAAm HG and PNIPAm-co-PAAm@Cur HG samples, respectively. Figure 2a shows the morphological appearance of the PNIPAm-co-PAAm HG and showed the aggregated particles with rough surfaces. This was because at 40 °C, the PNIPAm-co-PAAm HG forms an aggregated structure. Furthermore, the SEM image of the Cur-loaded PNIPAm-co-PAAm@Cur HG was measured. As observed in Figure 2b, the sample showed an irregular shape with some aggregated particles. The formation of aggregated particles may be caused by the conjugation of Cur molecules with the PNIPAm-co-PAAm HG sample.

### 3.2. pH-Responsive Behavior of PNIPAm-co-PAAm HG System

**Figure 3:**
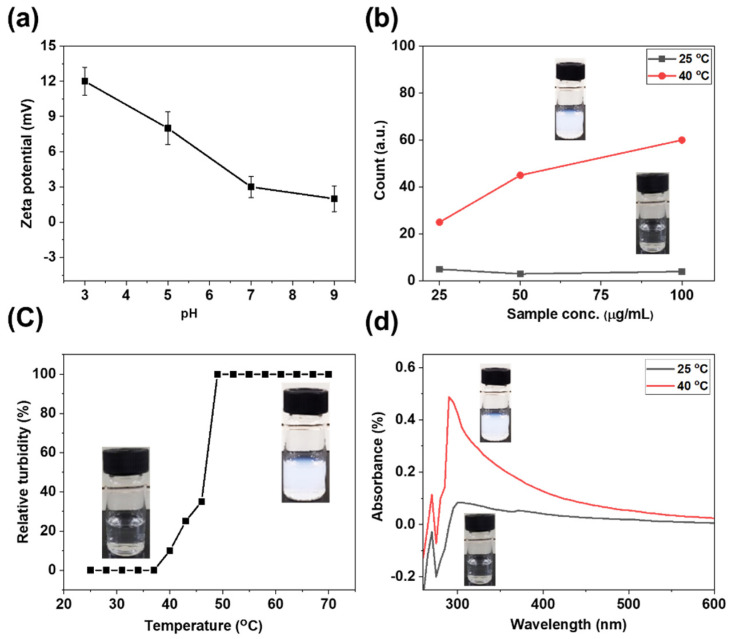
pH and thermo-responsive behavior of PNIPAm-co-PAAm HG. (**a**) Zeta potential and (**b**) particle size of PNIPAm-co-PAAm HG sample. (**c**) Relative turbidity of PNIPAm-co-PAAm HG sample (10 mg/mL) at 25 °C and 40 °C. (**d**) UV–vis absorption of PNIPAm-co-PAAm HG sample (10 mg/mL) at 25 °C and 40 °C.

The zeta potential was recorded to detect the magnitude of the electrical charge of PNIPAm-co-PAAm HG to study the swelling-deswelling behavior under pH stimulus. The zeta potential of the PNIPAm-co-PAAm HG system is pH sensitive due to the presence of carbonyl and amide groups. The zeta potential decreased from +12 to +2 mV, as the pH increased from 3 to 9. The hydrophobic part of PNIPAm segments in the PNIPAm-co-PAAm HG becomes aggregated into micelle cores at pH less than 5.5, and the hydrophilic PAAm segments are packed outside, forming the globule-like structure. PNIPAm-co-PAAm HG decreases protonation and increases hydrophilicity at high pH, allowing them to transit to the sol phase with linear copolymers (illustrated in Figure 3a,b). Dynamic light scattering (DLS) measurement was performed to verify the formation of linear to globule phase transformation at 40 °C. At 25 °C, there was a stable DLS count intensity for various sample concentrations, as shown in Figure 3b. Meanwhile, at 40 °C, the concentration-dependent DLS count intensity of the copolymer sample was observed. At 100 µg/mL, the DLS count intensity was nearly three times that of 25 µg/mL. The PNIPAm-co-PAAm HG sample transits to the turbid phase at temperatures above the LCST, increasing DLS count intensity; and higher sample concentrations resulted in higher DLS count intensity.

### 3.3. Thermo-Responsive Behavior of PNIPAm-co-PAAm HG System

Furthermore, the relative turbidity and UV–Vis absorption analysis of the prepared PNIPAm-co-PAAm HG were performed to investigate the swelling-deswelling behavior under the temperature stimulus of the PNIPAm-co-PAAm HG sample. The relative turbidity of the PNIPAm-co-PAAm HG (10 mg/mL) was measured from 25 to 70 °C, with an equilibrium time of 5 min at each temperature (Figure 3c). At the solution temperature of less than 40 °C, the sample became a homogeneous clear solution and almost 100% transparent. At a temperature below the LCST, the copolymer absorbs water and turns fully hydrated and remains in an expanded linear chain state. Starting at 40 °C, the transmittance solution turns turbid, and the samples’ relative turbidity increases greatly at 50 °C, indicating that the copolymer chains start to collapse into compact globule structures [33,34,35]. UV–Vis absorption of the PNIPAm-co-PAAm copolymer (10 mg/mL) was also measured at 25 °C and 40 °C, respectively. Because of the transparent and linear polymer structure, there was considerably less UV–Vis absorbance intensity observed at 25 °C. However, at 40 °C, temperature-induced micelle formation was significant due to turbidity caused by the formation of hydrophobic globule structures (Figure 3d).

### 3.4. Swelling–Deswelling Behavior and Phase Transition Mechanism of PNIPAm-co-PAAm HG

The most remarkable feature of dual pH- and thermosensitive hydrogels are that they can undergo phase transition under preferably a physical stimulus rather than a chemical stimulus. At low temperatures, the PNIPAm-co-PAAm HG sample is readily soluble in water and forms a homogeneous solution in a non-crosslinked form. PNIPAAm’s hydrophobic block promotes the aggregation of PNIPAm-co-PAAm HG. Theoretically, an amphiphilic copolymer forms hydrophobic domains and induced aggregation in the presence of hydrophilic polymer segments, due to the inter-polymer interactions becoming dominant. Recently, a micellar polymerization technique was used to create a hydrophilic monomer and a small number of hydrophobic segments. Along with the change in ionic interactions, hydrogen bonding also plays a crucial role in the phase transition, for example, poly(acrylamide).

**Figure 4:**
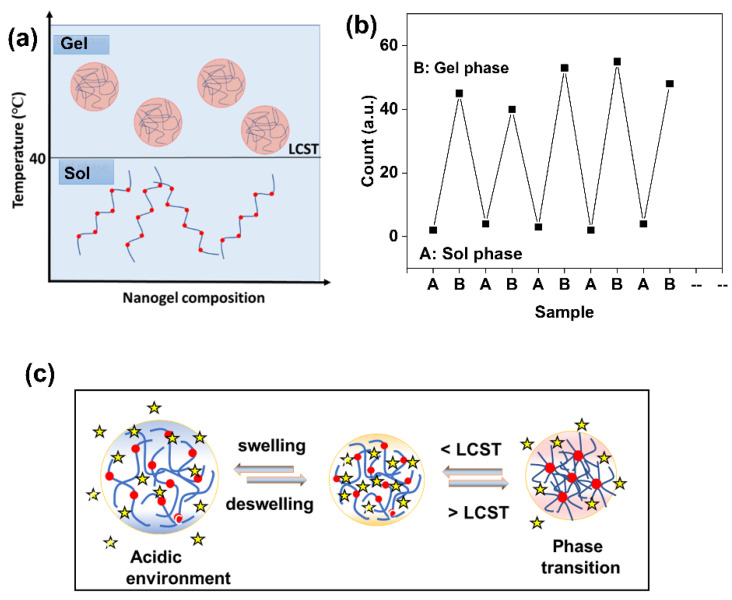
(**a**) A proposed schematic of the PNIPAm-co-PAAm HG conformational change in each phase state. Red color indicates the hydrophobic domain, and blue line indicates the hydrophilic polymer segments in the presence of water. (**b**) DLS analysis of PNIPAm-co-PAAm HG at below and above LCST. (**c**) Illustrates the phase transition of PNIPAm-co-PAAm HG under the pH stimulus. Red color indicates the hydrophobic domain, and blue line indicates the hydrophilic polymer segments in the presence of water.

PNIPAm-co-PAAm HG has an LCST of 40 °C and is composed of both amide (-CONH-) groups (hydrophilic) and isopropyl (-CH(CH_3_)_2_) side chains (hydrophobic). At around 32 °C, the PNIPAm HG undergoes a sharp phase transition in water. When the copolymer is formed with PAAm, the LCST of the PNIPAm-co-PAAm HG is around 40 °C. As a result, PNIPAm-co-PAAm HG solutions have a linear hydrophilic polymer chain (sol state) at RT and can be transformed into a hydrophobic globule coil state, when temperatures rise above 40 °C (Figure 4a). The temperature-responsive phase transition of the PNIPAm-co-PAAm HG was evaluated using the dynamic light scattering analysis. As shown in Figure 4b, there are no considerable particles formed for the PNIPAm-co-PAAm HG measured at 25 °C. On the other hand, micro sized particles were observed when the PNIPAm-co-PAAm HG was measured at 40 °C. This indicated that the PNIPAm-co-PAAm HG undergoes phase transition at above the LCST.

Because the LCST of the PNIPAm-co-PAAm HG is higher than that of the human body, quick micelle formation of these copolymers can be avoided when the PNIPAm-co-PAAm HG is injected into the body. Maintaining fine temperature control allows for the selective release of drugs at target sites in a controlled manner. This results in a well-organized structure in the solution that can transport and encapsulate hydrophobic drugs. The Cur drug can be easily encapsulated into the PNIPAm-co-PAAm HG for delivery by first dissolving the Cur at a lower temperature and then raising the temperature to above the LCST for micelle formation. The low temperature used to mix copolymers and Cur protects the Cur from denaturation or aggregation. The attractive ionic interactions cause the pH-based phase transition of the PNIPAm-co-PAAm HG (Figure 4c). The amide and carbonyl groups of the PNIPAm-co-PAAm HG attract the H^+^ ions. The polymer chains aggregate, and the attached functional groups covalently crosslink with water when the PNIPAm-co-PAAm HG is in the water. PNIPAm-co-PAAm HG ionizes at low pH, allowing the drug to be released into the low pH microenvironment of tumors (Figure 4c).

### 3.5. In Vitro Delivery of Cur from PNIPAm-co-PAAm@Cur HG System

**Figure 5:**
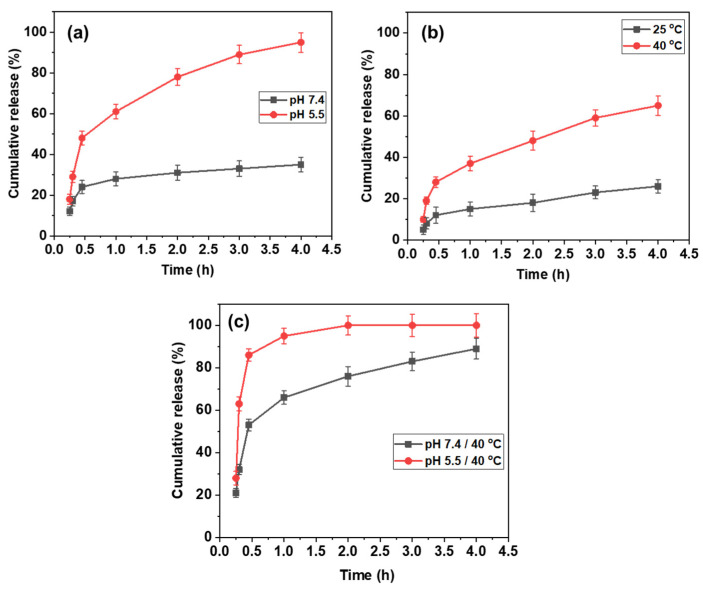
In vitro Cur delivery of PNIPAm-co-PAAm@Cur HG system. (**a**) Cur release with pH stimulus, (**b**) Cur release with temperature stimulus, and (**c**) Cur release with pH and temperature stimulus.

**Scheme 2:**
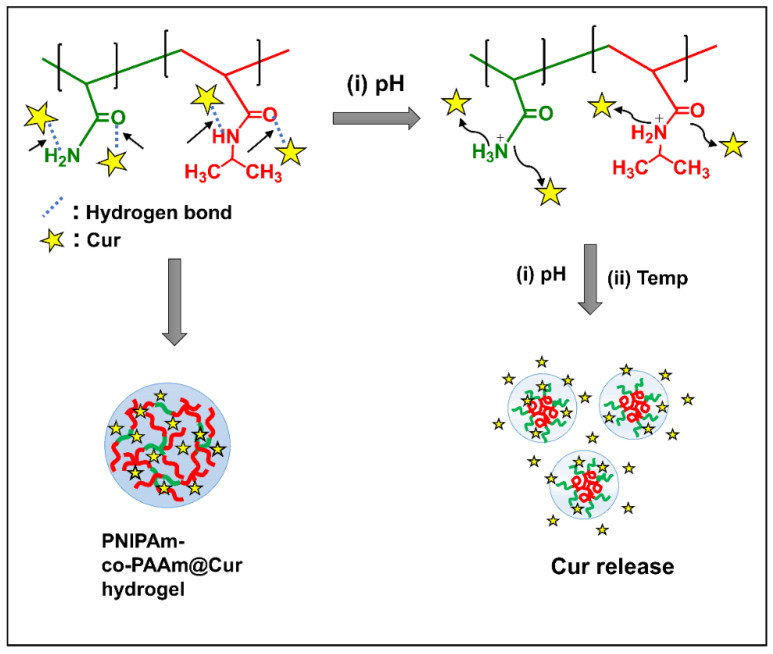
Schematic representation of drug loading and pH and temperature- responsive release behavior from PNIPAm-co-PAAm HG system.

The Cur release profiles from the Cur-loaded PNIPAm-co-PAAm@Cur HG system were measured at different conditions (pH 7.4 and pH 5.5; at 25 °C and 40 °C; at pH 7.4/40 °C and pH 5.5/40 °C) for competitive study. First, we tested the pH stimuli-responsive release of Cur at pH 7.4 and 5.5. As shown in Figure 5a, approximately 62% was released at pH 7.4 and about 92% of Cur was released at pH 5.5, respectively, in 4 h. The enhanced release behavior of Cur was due to the protonation of amine and amide part of the PNIPAm-co-PAAm@Cur HG system under acidic pH conditions. Similarly, the Cur release was gradually increased from 25% at 25 °C to 65% release at 40 °C in the 4 h release time (Figure 5b). About 65% of release at 40 °C might be due to the release of physically encapsulated Cur molecules in the PNIPAm-co-PAAm@Cur HG system. Furthermore, the Cur release efficiency of the PNIPAm-co-PAAm@Cur system was measured at pH 7.4/40 °C and pH 5.5/40 °C, respectively. The Cur was quickly released after 30 min at pH 5.5/40 °C, achieving 84% Cur release. At pH 5.5/40 °C, approximately 92% of the Cur was released after 1 h and nearly 100% of the Cur was released after 2 h. Meanwhile, only 32% was released after 30 min at pH 7.4/40 °C. At physiological pH (pH 7.4) conditions, the loaded drug can interact with amine and amide groups via H-bonding interactions, and only the weakly adhered drug molecules are released with a minimum amount of Cur release at pH 7.4/40 °C. The release efficiency increased gradually over time, reaching around 82% after 4 h. However, the Cur release was considerably enhanced under acidic pH (pH 5.5) conditions, due to the protonation of amine and amide groups and the Cur molecules, which induced an electrostatic repulsion that caused the release of the Cur from the system at pH 5.5. Furthermore, the Cur release was greatly enhanced in the presence of the combined acidic pH (5.5) and temperature (40 °C) (Figure 5c). This might be due to the acid-induced protonation and temperature-induced phase transition (chain structure to globule structure,) which push out the encapsulated drug molecules (Scheme 2). We conclude that dual stimuli such as pH and temperature resulted in the highest efficiency of Cur release from the PNIPAm-co-PAAm@Cur HG system, as compared to the individual stimuli (either pH or temperature). Several types of doping materials have been employed in the literature (Table 1 and Table 2) for the loading/release of various drugs, including Cur; however, thermoresponsive PNIPAm-based polymers have advantages, such as the capability to release drugs under physiological and external temperature stimulation circumstances [36,37,38,39,40]. The majority of the studies on thermoresponsive copolymers described in the literature [41,42,43,44] have focused on thermoresponsive behavior; however, the suggested PNIPAm-co-PAAm HG system functions as a dual pH and temperature-responsive cur-cumin delivery system.

### 3.6. In Vitro Cytotoxicity (MTT Assay) Study

**Figure 6:**
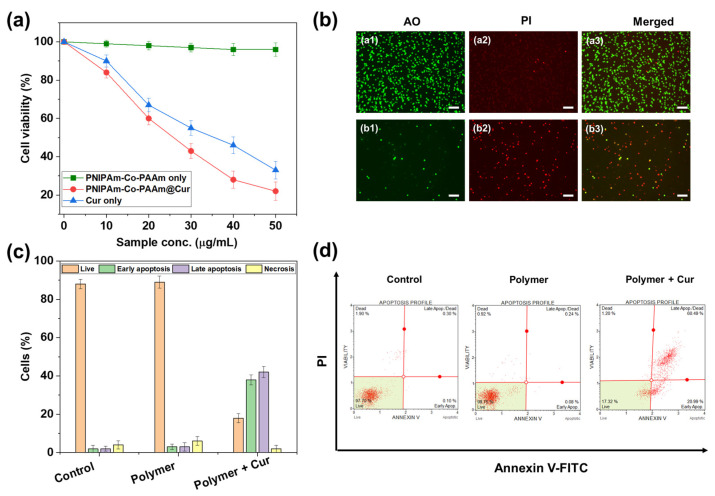
In vitro cytotoxicity of PNIPAm-co-PAAm@Cur HG system at 37 °C: (**a**) % cell viability. (**b**) Fluorescence microscopic images represent in vitro cytotoxicity of PNIPAm-co-PAAm@Cur HG. (**c**) and (**d**) demonstrate flow cytometry (FACS) analysis of PNIPAm-co-PAAm@Cur HG sample at 37 °C.

The in vitro biocompatibilities of the prepared PNIPAm-co-PAAm HG without and with Cur loading, respectively, were examined at 37 °C using a HepG2 cell line. As shown in Figure 6a, the PNIPAm-co-PAAm HG showed about ~90% viability to the HepG2 cells. In contrast, the Cur-loaded PNIPAm-co-PAAm@Cur HG sample showed concentration-dependent toxicity to the HepG2 cells. As shown in Figure 6a, the PNIPAm-co-PAAm@Cur HG sample loaded with Cur had higher cell viability than the pure Cur alone at equivalent concentrations of Cur at all tested concentrations; this was due to the lower Cur release from the PNIPAm-co-PAAm@Cur HG system at 37 °C compared to only Cur. This result matched the cumulative Cur release profiles. The MTT assay results support the conclusion that the prepared PNIPAm-co-PAAm HG is biocompatible and could be applied to the loading and release of anticancer drugs under tumor microenvironments.

### 3.7. Fluorescence Microscopic Images

The Cur drug-induced cell killing was also confirmed by fluorescence microscopy. The cells were stained with acridine orange (AO) and propidium iodide (PI) to distinguish the live and dead cells, respectively (Figure 6b). The HepG2 cells treated with only the PNIPAm-co-PAAm HG showed strong green fluorescence, indicating that most of the cells were live. However, the Cur-loaded PNIPAm-co-PAAm@Cur HG-treated cells showed a red color, suggesting that almost ~90% of the cells were dead. This might be due to the release of loaded Cur molecules that interact with the cells and cause death.

### 3.8. FACS Analysis

The biocompatibilities of the prepared PNIPAm-co-PAAm HG and Cur-loaded PNIPAm-co-PAAm@Cur HG system, respectively, were further examined by flow cytometry (Figure 6c,d). HepG2 cells were treated with the PNIPAm-co-PAAm HG and Cur-loaded PNIPAm-co-PAAm@Cur HG sample, respectively, for 4 h, and then the cells were stained with annexin V and PI stains. Dual-stained cells were indicated as late apoptosis and necrotic cells. As compared to the cells treated with the PNIPAm-co-PAAm HG sample (~15%), the Cur-loaded PNIPAm-co-PAAm@Cur HG sample increased the number of cells in early/late apoptosis (~85%) (Figure 6c). The observed results further support the conclusion that the prepared PNIPAm-co-PAAm HG is biocompatible and could be applied to the delivery of anticancer drugs in cancer therapy.

## 4. Conclusions

In summary, we prepared a pH and thermo-responsive PNIPAm-co-PAAm HG system for pH and temperature-triggered controlled release of anticancer drugs. Cur was used as a model anticancer drug to determine the loading and release efficiency of the PNIPAm-co-PAAm HG and the prepared HG system showed about ~65% Cur loading capacity. The PNIPAm-co-PAAm HG showed almost complete release of the loaded drug under both acidic pH (pH 5) and temperature (40 °C) stimuli conditions. Furthermore, the MTT assay and FACS results evidenced that the prepared PNIPAm-co-PAAm HG is biocompatible, and the loaded drug could be released under the intracellular microenvironment. The present study results evidenced that the PNIPAm-co-PAAm HG system has the potential to be useful in the delivery of antitherapeutic agents in cancer therapy. 

## Figures and Tables

**Table 1:** Comparison of PNIPAm-co-PAAm hydrogel and other doping materials.

Materials	Drug Delivery Stimuli	References
Mesoporous silica NPs	-	[36]
Organic porous polymer	-	[37]
Poly(L-histidine)-based nanovehicles	-	[38]
Mesoporous magnetic core and thermo-responsive shell for co-delivery of doxorubicin and curcumin	Temperature	[39]
Thermo-responsive alginate-g-Poly(N-isopropylacrylamide)	Temperature	[40]
PNIPAm-co-PAAm copolymer hydrogel	Dual pH and temperature	This paper

**Table 2:** Different design of PNIPAM-based copolymers for Cur delivery.

Polymers	Stimuli for Cur Delivery	References
Rechargeable pNIPAM hydrogel	Temperature	[41]
Chitosan-based hybrid PNIPAM hydrogel	Temperature	[42]
Thermoresponsive peptide-PNIPAm hydrogel	Temperature	[43]
PnBA-b-PNIPAM-b-PDMAEA triblock terpolymers	Temperature	[44]
PNIPAm-co-PAAm copolymer hydrogel	Dual pH and temperature	This paper

## Data Availability

Not applicable.
